# Predictive value of lymphocyte-to-monocyte ratio in the preoperative setting for progression of patients with breast cancer

**DOI:** 10.1186/s12885-018-5051-9

**Published:** 2018-11-19

**Authors:** Wataru Goto, Shinichiro Kashiwagi, Yuka Asano, Koji Takada, Katsuyuki Takahashi, Takaharu Hatano, Tsutomu Takashima, Shuhei Tomita, Hisashi Motomura, Kosei Hirakawa, Masaichi Ohira

**Affiliations:** 1 Department of Breast and Endocrine Surgery, Osaka City University Graduate School of Medicine, 1-4-3 Asahi-machi, Abeno-ku, Osaka, 545-8585 Japan; 2 Department of Pharmacology, Osaka City University Graduate School of Medicine, 1-4-3 Asahi-machi, Abeno-ku, Osaka, 545-8585 Japan; 3 Department of Plastic and Reconstructive Surgery, Osaka City University Graduate School of Medicine, 1-4-3 Asahi-machi, Abeno-ku, Osaka, 545-8585 Japan

**Keywords:** Lymphocyte-to-monocyte ratio, Prognostic marker, Breast cancer, Neutrophil-to-lymphocyte ratio, Neoadjuvant chemotherapy

## Abstract

**Background:**

The lymphocyte-to-monocyte ratio (LMR) has been used as a parameter reflecting systemic inflammation in several tumors, and is reportedly associated with prognosis in cancer patients. In this study, we evaluated the predictive value of LMR for progression and chemosensitivity in breast cancer patients treated with preoperative chemotherapy.

**Methods:**

LMR was evaluated in 239 patients with breast cancer treated with neoadjuvant chemotherapy (NAC) with 5-fluorouracil, epirubicin, and cyclophosphamide, followed by weekly paclitaxel with or without trastuzumab, and subsequent curative surgery. The correlations between LMR and clinicopathological features, prognosis, and pathological complete response (pCR) rate of NAC were evaluated retrospectively. We also evaluated the predictive value of neutrophil-to-lymphocyte ratio (NLR), and compared the predictive values of LMR and NLR.

**Results:**

We set 6.00 as the cut-off level for LMR based on the receiver operating characteristic (ROC) curve. A total of 119 patients (49.8%) were classified in the high-LMR group and 120 (50.2%) were classified in the low-LMR group. The low-LMR group had significantly worse disease-free survival rate (DFS) in all patients (*p* = 0.005) and in triple-negative breast cancer patients (*p* = 0.006). However, there was no significant correlation between LMR and pCR. Multivariate analysis showed that low LMR was an independent risk factor for DFS (*p* = 0.008, hazard ratio = 2.245). However, there was no significant difference in DFS (*p* = 0.143, log-rank) between patients in the low- and high-NLR groups.

**Conclusions:**

LMR may be a useful prognostic marker in patients with breast cancer.

**Electronic supplementary material:**

The online version of this article (10.1186/s12885-018-5051-9) contains supplementary material, which is available to authorized users.

## Background

Breast cancer is the most common type of cancer in women. Although neoadjuvant chemotherapy (NAC) increases the options for breast-conserving surgery and reduces the risk of postoperative recurrence in patients with resectable breast cancer [1–4], recurrence and metastasis still remain major problems, especially in patients with advanced-stage disease [5]. The tumor node metastasis (TNM) staging system and its associated phenotypes are important and useful tools for predicting prognosis [6, 7]. In breast cancer, molecular subtypes also affect the prognosis. Patients with triple-negative breast cancer (TNBC) or human epidermal growth factor receptor-2 (HER2)-enriched breast cancer have high recurrence rates and poor prognosis [8]. However, it has been reported that not only the tumor characteristics but also the host inflammatory response are important for cancer progression [9].

Inflammation affects cancer progression, and a chronic systemic inflammatory response is involved in poor outcome in breast cancer patients [10]. Furthermore, the host immune system has been found to influence the clinical response to chemotherapy, and should thus be taken into account even during conventional chemotherapy treatment [11]. Systemic inflammatory markers such as neutrophil to lymphocyte ratio (NLR) and platelet lymphocyte ratio (PLR) have been reported as prognostic factors in various cancers [12, 13]. Furthermore, the lymphocyte-to-monocyte ratio (LMR), which also reflects the degree of systemic inflammation, has recently been reported to correlate with survival in various types of malignancies, such as diffuse large B cell lymphoma, colon cancer, esophageal carcinoma, lung cancer [14–17]. In this single-center, retrospective study, we aimed to evaluate LMR and NLR as a possible marker for predicting the outcome of NAC in a consecutive series of patients with breast cancer treated with a standardized protocol.

## Methods

### Patient background

A total of 239 patients with resectable, early-stage breast cancer diagnosed as stage IIA (T1, N1, M0 or T2, N0, M0), IIB (T2, N1, M0 or T3, N0, M0), or IIIA (T1–2, N2, M0 or T3, N1–2, M0) were treated with NAC between 2007 and 2015. Tumor stage and T and N factors were stratified based on the TNM Classification of Malignant Tumors, UICC Seventh Edition [18]. Breast cancer was confirmed histologically by core needle biopsy and staged by systemic imaging studies using computed tomography, ultrasonography, and bone scintigraphy. Tumors were classified into subtypes according to the immunohistochemical expression of estrogen receptor, progesterone receptor, HER2, and Ki67. The cut-offs for estrogen receptor and progesterone receptor positivity were both > 0% positive tumor cells with nuclear staining. Tumors with 3+ HER2 on immunohistochemical staining were considered to show HER2 overexpression, tumors with 2+ HER2 were analyzed further by fluorescence in situ hybridization, and tumors with HER2/Centromere (CEP) 17 ≥ 2.0 were also considered to exhibit HER2 overexpression [19, 20]. A Ki67-labeling index ≥14% tumor cells with nuclear staining was determined to be positive [21].

All patients received a standardized NAC protocol consisting of four courses of FEC100 (500 mg/m^2^ fluorouracil, 100 mg/m^2^ epirubicin, and 500 mg/m^2^ cyclophosphamide) every 3 weeks, followed by 12 courses of 80 mg/m^2^ paclitaxel administered weekly [22, 23]. Sixty-eight patients had HER2-positive breast cancer and were additionally administered weekly (2 mg/kg) or tri-weekly (6 mg/kg) trastuzumab during paclitaxel treatment [24]. All patients underwent chemotherapy as outpatients. Therapeutic anti-tumor effects were assessed according to the Response Evaluation Criteria in Solid Tumors (RECIST) criteria [25]. Pathological complete response (pCR) was defined as the complete disappearance of the invasive compartment of the lesion with or without intraductal components, including the lymph nodes [1]. Patients underwent mastectomy or breast-conserving surgery after NAC. All patients who underwent breast-conserving surgery were administered postoperative radiotherapy to the remnant breast. Overall survival (OS) time was the period from the surgery to the time of death from any cause. Disease-free survival (DFS) was defined as freedom from all local, loco-regional, and distant recurrences. All patients were followed up by physical examination every 3 months, ultrasonography every 6 months, and computed tomography and bone scintigraphy annually. The median follow-up period for the assessment of OS was 3.7 years (range, 0.2–6.0 years) and for DFS was 3.4 years (range, 0.1–6.0 years).

### Ethics statement

This study was conducted at Osaka City University Graduate School of Medicine, Osaka, Japan, according to the Reporting Recommendations for Tumor Marker Prognostic Studies (REMARK) guidelines and following a retrospectively written research, pathological evaluation, and statistical plan [26]. This research conformed to the provisions of the 2013 Declaration of Helsinki. All patients were informed of the investigational nature of this study and provided their written informed consent. The Ethics Committee of Osaka City University approved the study protocol (#926).

### Blood sample analysis

Peripheral blood samples were obtained at the time of diagnosis, before the initiation of NAC. These were taken only once. The numbers of white blood cells were determined using a hemocytometer. The percentages of different cell types were determined using a Coulter LH 750 Hematology Analyzer (Beckman Coulter, Brea, CA, USA). LMR was calculated from the pretreatment blood sample by dividing the absolute lymphocyte count by the absolute monocyte count. NLR was calculated from the pretreatment blood sample by dividing the absolute neutrophil count by the absolute lymphocyte count. All patients had no self-reported acute infections or hematologic disorders.

### Statistical analysis

Statistical analysis was performed using the JMP11 software program (SAS Institute, Cary, NC, USA). Receiver operating characteristic (ROC) curve analysis was performed to select the most appropriate cut-off values for LMR and NLR, to stratify patients at high risk of malignancy-related recurrences. The optimal cut-off value was established by means of Youden’s index. Associations between LMR, NLR, and clinicopathological variables, and the significance of different prognostic markers were analyzed using χ^2^ or Fisher’s exact test or Mann-Whitney U test, as appropriate. OS and DFS were estimated using the Kaplan–Meier method and compared using the log-rank test. Univariate and multivariate hazard ratios (HRs) were computed for the study parameters with 95% confidence intervals (CIs) using a Cox proportional hazards model, and used in a backward stepwise method selecting lymph node status, pathological response, and LMR for variable selection in multivariate analyses. A *p* value < 0.05 was considered significant.

## Results

Clinical responses (pCR + partial response) were observed in 209 patients (87.5%, 209/239). NAC-related pCR was observed in 91 patients (38.1%, 91/239). The pCR rates were 48.2% (40/83) and 32.7% (51/156) in patients with TNBC and non-TNBC, respectively (Table 1). Among all cases, DFS was significantly better in the pCR group compared with the non-pCR group (*p* = 0.040) (Additional file 1: Figure S1a), while OS tended to be better in the pCR group (*p* = 0.058) (Additional file 1: Figure S1b).

**Table 1:** Clinical response rate and pathological response rate to neoadjuvant chemotherapy

pathological response	all breast cancer (*n* = 239)	TNBC q(*n* = 83, 34.7%)	non-TNBC (*n* = 156, 65.3%)
pCR: pathological complete response			
CR: complete response	91 (38.1%)	40 (48.2%)	51 (32.7%)
non-pCR: non-pathological complete response			
PR: partial response	118 (49.4%)	34 (41.0%)	84 (53.8%)
SD: stable disease	25 (10.5%)	7 (8.4%)	18 (11.5%)
PD: progressive disease	5 (2.0%)	2 (2.4%)	3 (2.0%)
RR (CR + PR):response rate	209 (87.5%)	74 (89.2%)	135 (86.5%)

*TNBC* triple-negative breast cancers

**Fig. 1:**
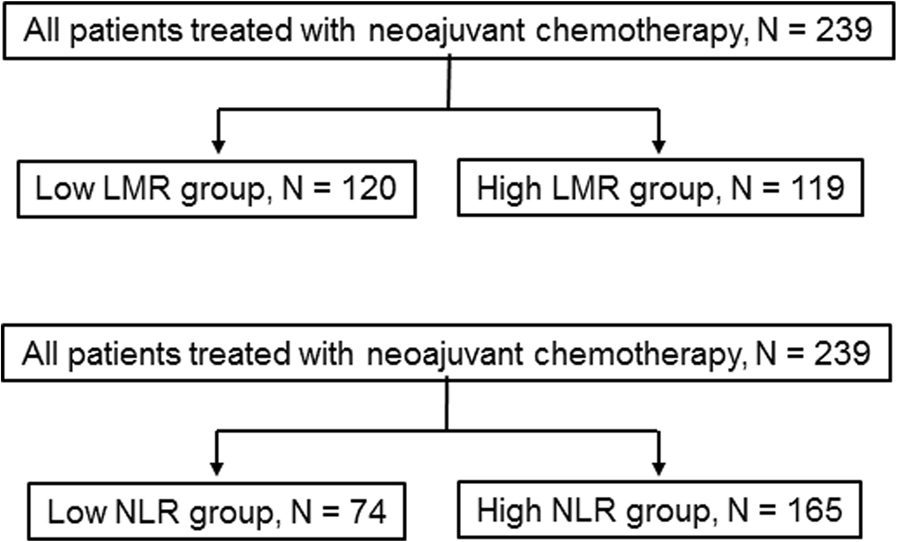
Flowchart of patient selection

LMR was determined in every sample and ranged from 1.8–15.2 (mean, 6.2; median, 5.9; standard deviation, 2.3). The LMR cut-off value for DFS was 6.00 (area under the curve (AUC): 0.57; sensitivity: 61.3%; specificity: 59.2%) (Additional file 2: Figure S2a). We therefore classified patients into low-LMR (*n* = 120, 50.2%) and high-LMR (*n* = 119, 49.8%) groups according to a cut-off value of 6.0 (Fig. 1). LMR was significantly correlated with age (*p* = 0.004), menopausal status (*p* = 0.008), and tumor size (*p* = 0.036). There was no significant correlation between LMR and any other tested clinicopathological parameter, including pCR (Table 2). In continuous variables, older age (*p* < 0.001), menopause (*p* < 0.001), and higher Ki-67 (*p* = 0.016) were significantly associated with higher LMR (Additional file 3: Figure S3). NLR was also determined in every sample, and ranged from 0.5–10.6 (mean, 2.3; median, 2.0; standard deviation, 1.3). The NLR cut-off value for DFS was 1.63 (AUC: 0.56; sensitivity: 75.6%; specificity: 36.7%) (Additional file 2: Figure S2b). We therefore classified patients into low-NLR (*n* = 74) and high-NLR (*n* = 165) groups according to a cut-off value of 1.63 (Fig. 1). NLR was significantly correlated with age (*p* < 0.001) and menopausal status (*p* < 0.001), but there was no significant correlation between NLR and any other tested clinicopathological parameter, including pCR. In continuous variables, older age (*p* = 0.011) and menopause (*p* = 0.008) were significantly associated with lower NLR (Additional file 4: Figure S4).

**Table 2:** Correlation between clinicopathological features and NLR and LMR in 239 all breast cancers

Parameters	NLR		*p* value	LMR		*p* value
	High (*n* = 165, 69.0%)	Low (*n* = 74, 31.0%)		High (*n* = 119, 49.8%)	Low (*n* = 120, 50.2%)	
Age at operation						
≤ 56	95 (57.6%)	22 (29.7%)	< 0.001	47 (39.5%)	70 (58.3%)	0.004
> 56	70 (42.4%)	52 (70.3%)		72 (60.5%)	50 (41.7%)	
Menopause						
Pre-	78 (47.3%)	17 (23.0%)	< 0.001	37 (31.1%)	58 (48.3%)	0.008
Post-	87 (52.7%)	57 (77.0%)		82 (68.9%)	62 (51.7%)	
Tumor size						
≤ 2 cm	23 (13.9%)	9 (12.2%)	0.838	10 (8.4%)	22 (18.3%)	0.036
> 2 cm	142 (86.1%)	65 (87.8%)		109 (91.6%)	98 (81.7%)	
Lymph node status						
Negative	42 (25.5%)	24 (32.4%)	0.277	36 (30.3%)	30 (25.0%)	0.388
Positive	123 (74.5%)	50 (67.6%)		83 (69.7%)	90 (75.0%)	
Nuclear grade						
1, 2	132 (80.0%)	63 (85.1%)	0.373	99 (82.5%)	96 (80.7%)	0.741
3	33 (20.0%)	11 (14.9%)		21 (17.5%)	23 (19.3%)	
Ki67						
≤ 14%	53 (32.1%)	26 (35.1%)	0.658	35 (29.4%)	44 (36.7%)	0.272
> 14%	112 (67.9%)	48 (64.9%)		84 (70.6%)	76 (63.3%)	
Intrinsic subtype						
TNBC	60 (36.4%)	23 (31.1%)	0.465	40 (33.6%)	43 (35.8%)	0.719
Non-TNBC	105 (63.6%)	51 (68.9%)		79 (66.4%)	77 (64.2%)	
Pathological response						
pCR	69 (41.8%)	22 (29.7%)	0.085	46 (38.3%)	45 (37.8%)	0.934
non-pCR	96 (58.2%)	52 (70.3%)		74 (61.7%)	74 (62.2%)	

*TNBC* triple-negative breast cancer, *pCR* pathological complete response, *NLR* neutrophil-to-lymphocyte ratio, *LMR* lymphocyte-to-monocyte ratio

**Fig. 2:**
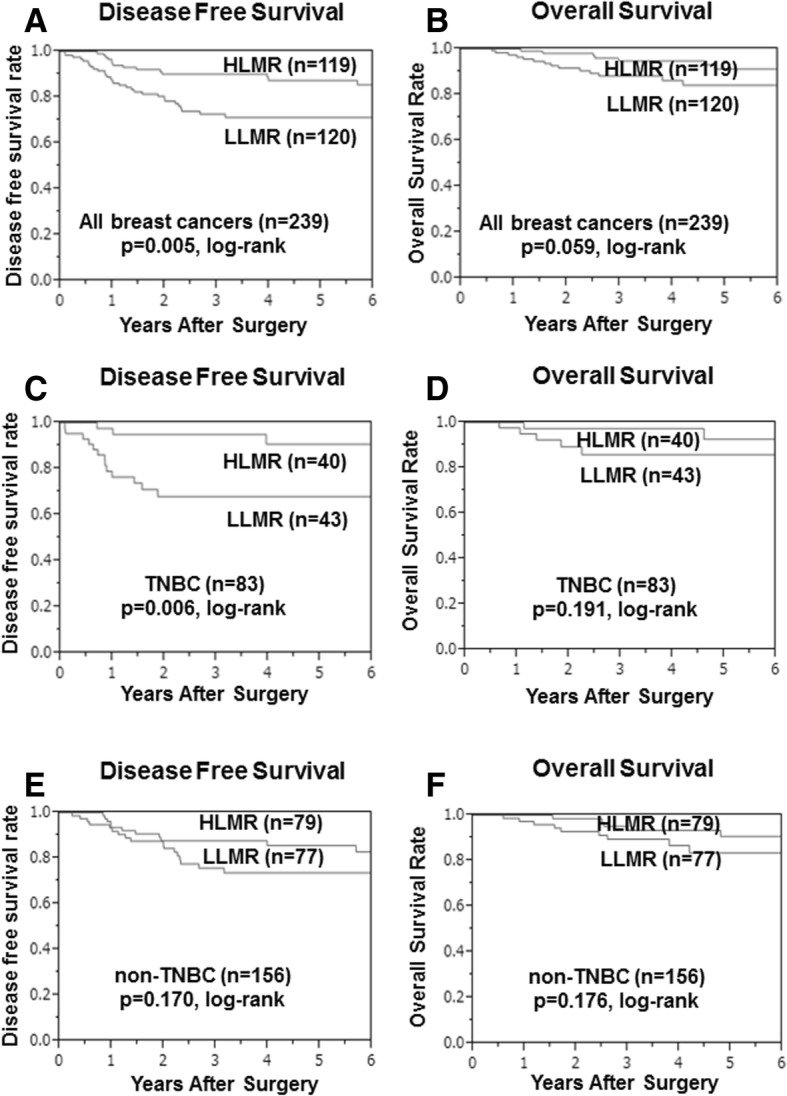
Survival was analyzed according to LMR. DFS was significantly worse in the low- compared with the high-LMR group (*p* = 0.005) (**a**). OS tended to be worse in the low- compared with the high-LMR group (*p* = 0.059) (**b**). Among 83 TNBC patients, DFS was significantly longer (*p* = 0.006) in the high- compared with the low-LMR group (**c**), but OS was not significantly different (*p* = 0.059) (**d**). Among 156 TNBC patients, there was no significant difference between the low- and high-LMR groups for DFS (*p* = 0.170) (**e**) or OS (*p* = 0.176) (**f**)

**Fig. 3:**
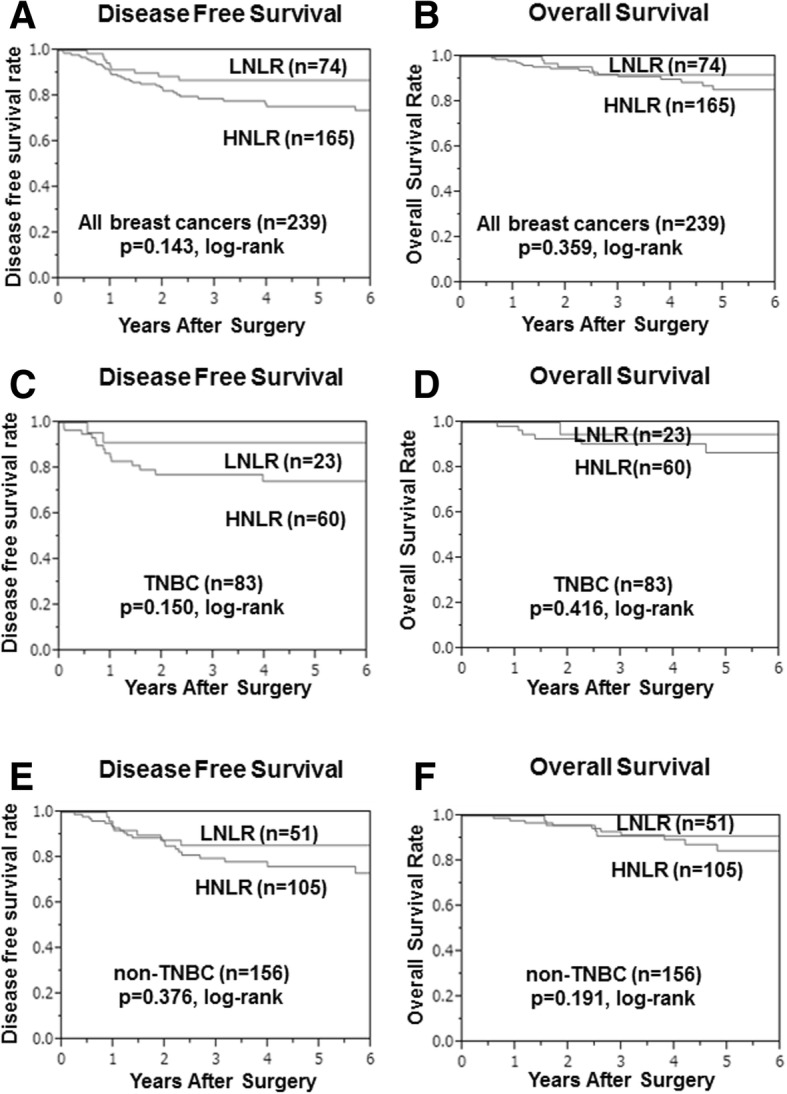
Survival was analyzed according to NLR. There were no significant differences between the low- and high-NLR groups for DFS or OS in all breast cancers (*p* = 0.143) (**a**) (*p* = 0.359) (**b**), TNBC (*p* = 0.150) (**c**) (*p* = 0.416) (**d**), and non-TNBC patients (*p* = 0.376) (**e**) (*p* = 0.191) (**f**)

DFS was significantly worse in the low- compared with the high-LMR group (*p* = 0.005) (Fig. 2a), while OS tended to be worse in the low-LMR group (*p* = 0.059) (Fig. 2b). Among 83 TNBC patients, DFS was significantly longer (*p* = 0.006) in the high- compared with the low-LMR group (Fig. 2c), but OS was not significantly different (*p* = 0.191) (Fig. 2d). Among 156 non-TNBC patients, there was no significant difference between the low- and high-LMR groups in terms of DFS (*p* = 0.170) (Fig. 2e) or OS (*p* = 0.176) (Fig. 2f). There were no significant differences between the low- and high-NLR groups in terms of DFS or OS for all breast cancers (*p* = 0.143 and *p* = 0.359, respectively) (Fig. 3a,b), TNBC (*p* = 0.150 and *p* = 0.416, respectively) (Fig. 3c,d), and non-TNBC patients (*p* = 0.376 and *p* = 0.191, respectively) (Fig. 3e,f).

The correlations between DFS and the various clinicopathological factors in 239 all breast cancers are shown in Table 3. According to the results of univariate analysis, DFS exhibited significant relationships with lymph node status (*p* = 0.020), complete response to chemotherapy (*p* = 0.034), and LMR (*p* = 0.005). In contrast, NLR was not a prognostic factor for DFS (*p* = 0.116). Multivariate analysis indicated that lymph node status (HR = 2.826, 95 %CI: 1.281–7.474, *p* = 0.008), and LMR (HR = 2.245, 95 %CI: 1.237–4.233, *p* = 0.008) were independent prognostic factors for survival (Table 3). Additionally, in 83 TNBC patients, multivariate analysis revealed that pathological response (HR = 2.921, 95 % CI: 1.015–10.470, *p* = 0.047) and LMR (HR = 4.675, 95 % CI: 1.500–20.445, *p* = 0.006) were significantly correlated with RFS (Additional file 5: Table S1).

**Table 3:** Univariate- and multivariate analysis with respect to disease-free survival in 239 all breast cancers

Parameter		Univariate analysis			Multivariate analysis		
		Hazard ratio	95% CI	*p* value	Hazard ratio	95% CI	*p* value
		all breast cancers (*n* = 239)					
Age	≤56	1.434	0.808–2.574	0.218			
Menopause	Pre	1.149	0.637–2.040	0.639			
Tumor size (cm)	> 2	1.799	0.728–5.979	0.223			
Lymph node status	Positive	2.494	1.143–6.550	0.020	2.826	1.281–7.474	0.008
Nuclear grade	3	1.086	0.510–2.103	0.819			
Ki67 (%)	≤14	1.717	0.959–3.052	0.069			
Intrinsic subtype	TNBC	1.015	0.542–1.828	0.962			
Pathological response	non-pCR	1.965	1.050–3.948	0.034	1.693	0.861–3.514	0.129
LMR	Low	2.318	1.285–4.350	0.005	2.245	1.237–4.233	0.008
NLR	High	1.680	0.884–3.465	0.116			

*TNBC* triple-negative breast cancer, *pCR* pathological complete response, *NLR* neutrophil-to-lymphocyte ratio, *LMR* lymphocyte-to-monocyte ratio, *CI* confidence interval

## Discussion

Inflammation and cancer show a strong association, and pretreatment levels of peripheral inflammatory cells, including neutrophils, lymphocytes and monocytes are reported as prognositic factors in various cancers [27–29]. Lymphocytes play an important role in host tumor immunity (for example, in cytotoxic cell death and inhibition of tumor cell proliferation and migration) [30–33]. Decreased lymphocyte numbers are therefore considered to be responsible for an insufficient immunologic reaction to the tumor, thus promoting tumor progression and metastasis [15]. Monocytes are known to infiltrate tumors and differentiate into tumor-associated macrophages, which are involved in tumor proliferation, invasion, metastasis, neovascularization, and recurrence [34, 35]. Increased levels of monocytes thus reflect a high tumor burden in patients with cancer. In such a mechanism, LMR is believed to reflect the host immune status and the degree of tumor progression. Given that both a low lymphocyte count and high monocyte count reflect insufficient anti-tumor immunity and an elevated tumor burden, a low LMR is therefore associated with a poorer prognosis.

In this study, we evaluated the predictive value of pre-NAC LMR and NLR for progression and chemosensitivity in breast cancer, and compared the predictive values of these systemic inflammatory markers, and showed that LMR was significantly associated with DFS in all breast cancer patients who received NAC. This result suggests that LMR may be one of the criteria for deciding whether to perform adjuvant chemotherapies with active regimens from the beginning.

There are many publications concerning the prognostic value of LMR or NLR in breast cancer [36–44]. The appropriate cut-off values for LMR and NLR were set by ROC curve in most studies, and were not unified. Recently, Ethier et al. conducted meta-analysis on the relationship between NLR and prognosis of breast cancer, and reported that the median cut-off value for NLR was 3.0 [45]. However, to our knowledge, no meta-analysis on the relationship between LMR and prognosis of breast cancer has been conducted. In addition, some studies evaluated the predictive value of LMR or NLR for progression and chemosensitivity in breast cancer patients treated with NAC, however, there are few reports to investigate the prognostic value of both LMR and NLR in breast cancer patients following NAC. Marín et al. analysed 150 breast cancer patients treated with NAC and reported that patients with high LMR and low NLR were associated with good DFS [44]. On the other hand, our study reported that only LMR was significantly correlated with prognosis. There are two possible reasons for this inconsistent result. First, the appropriate cut-off values for LMR and NLR differed in each study. Second, the standardized protocol of NAC differed in each study. In Marín’s study, NAC regimen was based on anthracyclines and taxanes ± trastuzumab, and pCR rate was 17.6%. In our study, NAC regimen was based on FEC100 followed by weekly paclitaxel ± trastuzumab, and pCR rate was higher than Marín’s study, 38.1%. 5-fluorouracil is shown to increase the sensitivity of cancer cells to killing by cytotoxic T cells (CTLs). In addition, a DNA alkylating agent cyclophosphamide can lead to improved T cell effector functions and stimulate an immunogenic death. Moreover, CTLs are effective against paclitaxel-treated tumor cells and induce tumor cell apoptosis [46, 47]. In other words, our regimen plays a role in enhancing the immune response.

Moreover, the present study is the first to evaluate the prognostic value of both LMR and NLR in different molecular subtypes of breast cancer following NAC. Weijuan et al. reported that decreased LMR was significantly associated with a poor prognosis for TNBC subtype in a study of 1570 patients [38]. The present study found no relationship between LMR and intrinsic subtype, but did reveal significant differences in DFS among all 239 patients, and among 83 TNBC patients stratified by LMR. TNBC is known to exhibit poor clinical outcomes compared with non-TNBC, and a recent study suggested that TNBC may be more strongly influenced by systemic inflammatory function [41].

Recently, it is becoming evident that the surrounding cancer microenvironment greatly influences cancer cells and plays a role in the development of characteristic cancer features [48]. Lymphocytes play not only as systemic inflammatory markers but also as tumor-infiltrating lymphocytes (TILs) that evaluate tumor immune responses [49–51], and monocytes also relate to tumor-associated macrophages. Matsumoto et al. reported that high levels of tumor-infiltrating CD8+ T-cells may reflect an improved prognosis in terms of chemotherapy sensitivity in TNBC, and that tumor-associated macrophages were associated with a relatively poor outcome in patients with TNBC [52]. We also examined the prognostic significance of TILs before NAC in same breast cancer patients [53]. As in present study, DFS was significantly longer in the high TILs group than in the low TILs group among all breast cancer and TNBC patients. These results suggested that local immune tumor microenvironment and systemic inflammation had relationship. From now on, by further evaluating tumor-infiltrating lymphocytes or tumor-associated macrophages, along with systemic inflammatory markers such as LMR or NLR, more accurate identification of patient-specific immune mechanisms and prediction of prognosis may be possible.

As a potential limitation, this study is a single-center retrospective study, and the sample size is small, and the numbers of TNBC patients are thus even smaller. Further prospective multicenter studies are therefore needed to identify the strengths and weaknesses of our findings.

## Conclusions

The findings of this study indicate that pretreatment LMR is a useful prognostic marker in patients with breast cancer.

## Additional files

Additional file 1: **Figure S1.** Survival was analyzed according to pCR. Among breast cancer cases, DFS was significantly better in the pCR group compared with the non-pCR group (*p* = 0.040) (a) and OS tended to be better in the pCR group (*p* = 0.058) (b). (TIF 118 kb)

Additional file 2: **Figure S2**. ROC curve analyses of the LMR and NLR in breast cancer patients. The LMR cut-off value for DFS was 6.00 (AUC: 0.57335, sensitivity: 61.3%, specificity: 59.2%) (a). The NLR cut-off value for DFS was 1.63 (AUC: 0.56064, sensitivity: 75.6%, specificity: 36.7%) (b). (TIF 167 kb)

Additional file 3: **Figure S3.** Correlation between clinicopathological features and LMR in 239 all breast cancers in continuous variables. Older age (*p* < 0.001), menopause (*p* < 0.001), and higher Ki-67 (*p* = 0.016) were significantly associated with higher LMR. (TIF 141 kb)

Additional file 4: **Figure S4.** Correlation between clinicopathological features and NLR in 239 all breast cancers in continuous variables. Older age (*p* = 0.011) and menopause (*p* = 0.008) were significantly associated with lower NLR. (TIF 128 kb)

Additional file 5 **Table S1.** Univariate- and multivariate analysis with respect to disease-free survival in 83 triple-negative breast cancer. (DOCX 21 kb)

## Abbreviations

CI: Confidence interval
CTLs: Cytotoxic T cells
DFS: Disease-free survival
HER2: Human epidermal growth factor receptor-2
HR: Hazard ratio
LMR: Lymphocyte-to-monocyte ratio
NAC: Neoadjuvant chemotherapy
NLR: Neutrophil-to-lymphocyte ratio
OS: Overall survival
pCR: Pathological complete response
RECIST: Response Evaluation Criteria in Solid Tumors
REMARK: Reporting Recommendations for Tumor Marker Prognostic Studies
ROC: Receiver operating characteristic
TILs: Tumor-infiltrating lymphocytes
TNBC: Triple-negative breast cancer
TNM: Tumor node metastasis
