# Supplementary figures and images for: Predictive value of lymphocyte-to-monocyte ratio in the preoperative setting for progression of patients with breast cancer
Source: BMC Cancer. 2018 Nov 19;18:1137. doi: 10.1186/s12885-018-5051-9 (PMC6245848; doi:10.1186/s12885-018-5051-9)

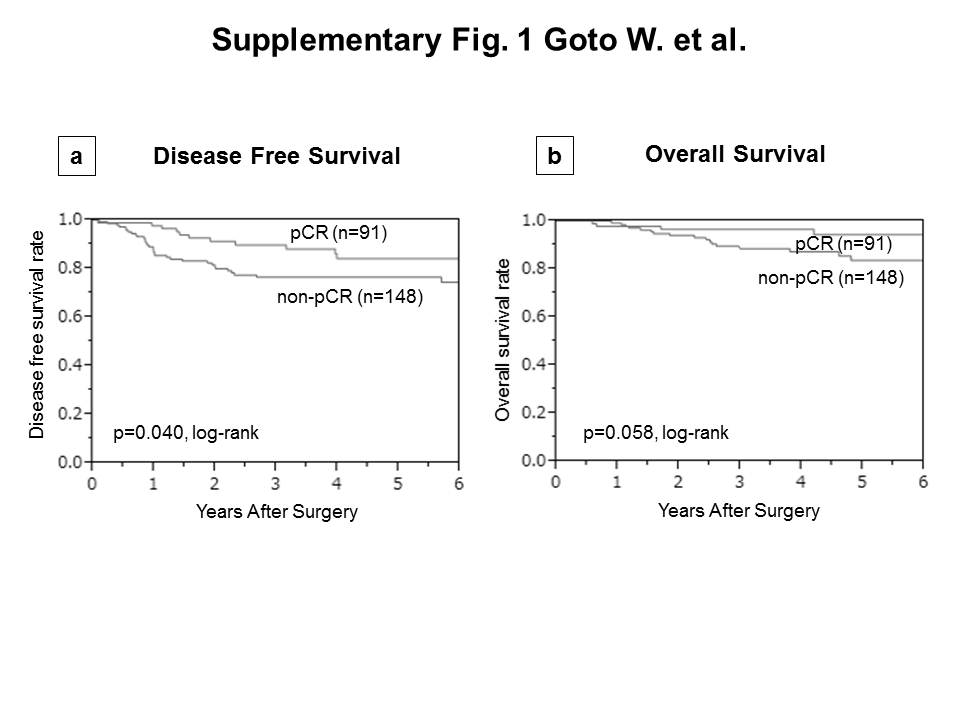

Supplement: Supplementary file 1 — Figure S1. Survival was analyzed according to pCR. Among breast cancer cases, DFS was significantly better in the pCR group compared with the non-pCR group (p = 0.040) (a) and OS tended to be better in the pCR group (p = 0.058) (b). (TIF 118 kb) [file 12885_2018_5051_MOESM1_ESM.tif]

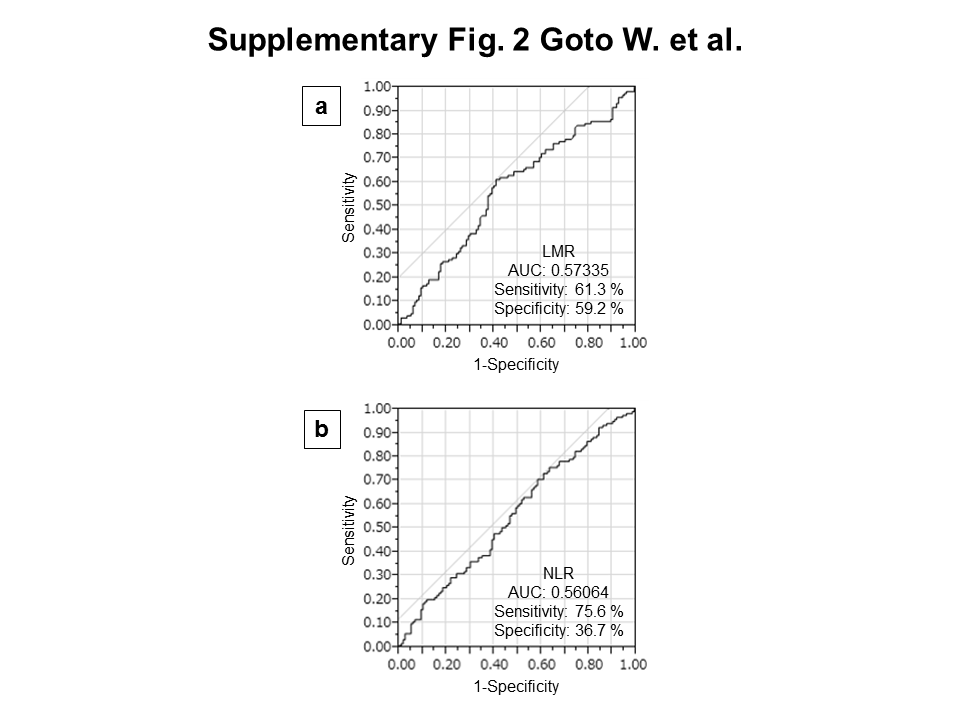

Supplement: Supplementary file 2 — Figure S2. ROC curve analyses of the LMR and NLR in breast cancer patients. The LMR cut-off value for DFS was 6.00 (AUC: 0.57335, sensitivity: 61.3%, specificity: 59.2%) (a). The NLR cut-off value for DFS was 1.63 (AUC: 0.56064, sensitivity: 75.6%, specificity: 36.7%) (b). (TIF 167 kb) [file 12885_2018_5051_MOESM2_ESM.tif]

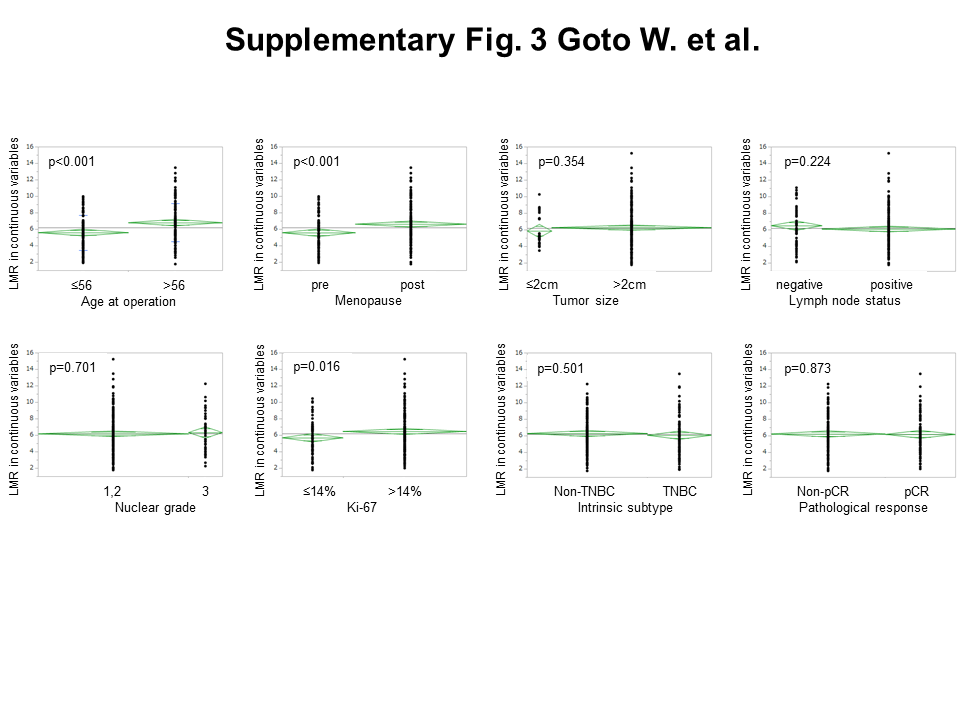

Supplement: Supplementary file 3 — Figure S3. Correlation between clinicopathological features and LMR in 239 all breast cancers in continuous variables. Older age (p < 0.001), menopause (p < 0.001), and higher Ki-67 (p = 0.016) were significantly associated with higher LMR. (TIF 141 kb) [file 12885_2018_5051_MOESM3_ESM.tif]

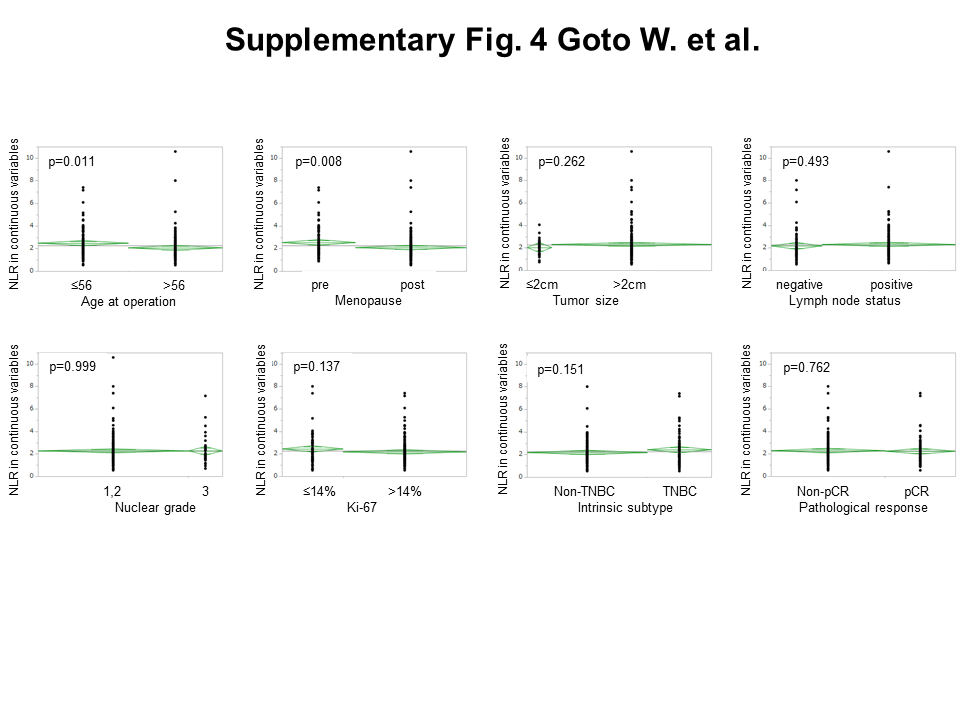

Supplement: Supplementary file 4 — Figure S4. Correlation between clinicopathological features and NLR in 239 all breast cancers in continuous variables. Older age (p = 0.011) and menopause (p = 0.008) were significantly associated with lower NLR. (TIF 128 kb) [file 12885_2018_5051_MOESM4_ESM.tif]
