# Supplementary material for: Predictive value of lymphocyte-to-monocyte ratio in the preoperative setting for progression of patients with breast cancer
Source: BMC Cancer. 2018 Nov 19;18:1137. doi: 10.1186/s12885-018-5051-9 (PMC6245848; doi:10.1186/s12885-018-5051-9)
Supplement: Supplementary file 5 — Table S1. Univariate- and multivariate analysis with respect to disease-free survival in 83 triple-negative breast cancer. (DOCX 21 kb) [file 12885_2018_5051_MOESM5_ESM.docx]

**Supplement Table 1. Univariate- and multivariate analysis with respect to disease-free survival in 83 triple-negative breast cancer.**

| Parameter | | Univariate analysis | | | | Multivariate analysis | | |
| --- | --- | --- | --- | --- | --- | --- | --- | --- |
|  |  | Hazard ratio | 95 % CI | p value |  | Hazard ratio | 95 % CI | p value |
|  |  | all breast cancers (n=239) | | | | | | |
| Age | ≤56 | 1.750 | 0.649-5.153 | 0.271 |  |  |  |  |
| Menopause | Pre | 1.298 | 0.464-3.488 | 0.607 |  |  |  |  |
| Tumor size (cm) | >2 | 0.803 | 0.232-5.288 | 0.810 |  |  |  |  |
| Lymph node status | Positive | 1.059 | 0.367-3.796 | 0.921 |  |  |  |  |
| Nuclear grade | 3 | 1.283 | 0.359-3.685 | 0.673 |  |  |  |  |
| Ki67 (%) | ≤14 | 1.443 | 0.454-3.972 | 0.508 |  |  |  |  |
| Pathological response | non-pCR | 3.167 | 1.102-11.339 | 0.032 |  | 2.921 | 1.015-10.470 | 0.047 |
| LMR | Low | 4.963 | 1.594-21.689 | 0.004 |  | 4.675 | 1.500-20.445 | 0.006 |
| NLR | High | 2.833 | 0.791-18.040 | 0.119 |  |  |  |  |

TNBC, triple-negative breast cancer. pCR, pathological complete response. NLR, neutrophil-to-lymphocyte ratio. LMR, lymphocyte-to-monocyte ratio. CI, confidence interval.
